# Magneto-Liposomes as MRI Contrast Agents: A Systematic Study of Different Liposomal Formulations

**DOI:** 10.3390/nano10050889

**Published:** 2020-05-06

**Authors:** Nina Kostevšek, Calvin C. L. Cheung, Igor Serša, Mateja Erdani Kreft, Ilaria Monaco, Mauro Comes Franchini, Janja Vidmar, Wafa T. Al-Jamal

**Affiliations:** 1 Department for Nanostructured Materials, Jožef Stefan Institute, 1000 Ljubljana, Slovenia; 2 School of Pharmacy, Queen’s University Belfast, Belfast BT9 7BL, UK; ccheung04@qub.ac.uk; 3 Condensed Matter Physics Department, Jožef Stefan Institute, 1000 Ljubljana, Slovenia; igor.sersa@ijs.si; 4 Faculty of Medicine, Institute of Cell Biology, University of Ljubljana, 1000 Ljubljana, Slovenia; mateja.erdani@mf.uni-lj.si; 5 Department of Industrial Chemistry “Toso Montanari”, University of Bologna, 40136 Bologna, Italy; ilaria.monaco@bio-on.it (I.M.); mauro.comesfranchini@unibo.it (M.C.F.); 6 Department for Environmental Sciences, Jožef Stefan Institute, 1000 Ljubljana, Slovenia; janja.vidmar@ijs.si

**Keywords:** liposomes, magnetic resonance imaging, iron oxide, contrast agent

## Abstract

The majority of the clinically approved iron oxide nanoparticles (IO NPs) used as contrast agents for magnetic resonance imaging (MRI) have been withdrawn from the market either due to safety concerns or lack of profits. To address this challenge, liposomes have been used to prepare IO-based *T*_2_ contrast agents. We studied the influence of different phospholipids on the relaxivity (*r*_2_) values of magneto-liposomes (MLs) containing magnetic NPs in the bilayer, where a strong correlation between the bilayer fluidity and *r*_2_ is clearly shown. Embedding 5-nm IO NPs in the lipid bilayer leads to a significant improvement in their relaxivity, where *r*_2_ values range from 153 ± 5 s^−1^ mM^−1^ for DPPC/cholesterol/DSPE-PEG (96/50/4) up to 673 ± 12 s^−1^ mM^−1^ for DOPC/DSPE-PEG (96/4), compared to “free” IO NPs with an *r*_2_ value of 16 s^−1^ mM^−1^, measured at 9.4 T MRI scanner. In vitro MRI measurements, together with the ICP-MS analysis, revealed MLs as highly selective contrast agents that were preferentially taken up by cancerous T24 cells, which led to an improvement in the contrast and an easier distinction between the healthy and the cancerous cells. A careful selection of the lipid bilayer to prepare MLs could offer efficient MRI contrast agents, even at very low IO NP concentrations.

## 1. Introduction

Magnetic resonance imaging (MRI) has good anatomic resolution and excellent soft-tissue contrast imaging capabilities. MRI contrast agents are used to enhance the image contrast between diseased and healthy tissues, thus improving MRI sensitivity and accuracy [1]. Gadolinium (Gd) chelates [2] and iron oxide nanoparticles (IO NPs) [3] are the most common clinical *T*_1_ and *T*_2_ contrast agents, respectively. Since the mid-1990s several iron-based MRI contrast agents have been developed. A few have been marketed worldwide under the commercial names listed in Table 1, where a list of all IO-based contrast agents with a description of the NP’s size, coating, and relaxivity values, together with the intended use and status on the market are shown. So far, only five IO NPs have been clinically approved for MRI imaging [4,5]. Unfortunately, commercial production of the majority of these contrast agents ceased due to commercial reasons (lack of sales), and one (Combidex^®^) was terminated in phase III of the clinical trials because it failed to show a significant improvement in contrast on the MR image [5]. Currently, only Resovist^®^ (carboxydextran-coated Fe_3_O_4_ NPs, available only in limited countries) and Feraheme^®^ (carbohydrate-coated Fe_3_O_4_ NPs, withdrawn from the EU market) are available [6]. The data in Table 1 clearly show that all the marketed IO-based contrast agents have relatively low relaxivities. Among them, the highest *r*_2_ value is shown by Resovist (189 mM^−1^ s^−1^), which is still on the market. Therefore, there is a need and a market space for novel IO NP-based imaging agents with a high safety margin and superior MRI properties. Liposomes are the most clinically approved nano-sized delivery systems [7], which proves their biocompatibility. Furthermore, their structure allows the encapsulation of additional active components in their aqueous core, offering a great platform for the preparation of multifunctional nanoparticles for theranostics and image-guided drug delivery, including MRI imaging.

Only a few reports have been published so far describing magneto-liposomes (MLs) as promising MRI contrast agents [11,12,13,14,15]. Interestingly, the majority of these studies reported that the encapsulation of hydrophilic IO NPs in the liposomes’ aqueous core (so-called H-MLs) reduced particle aggregation under physiological conditions, enhanced tissue accumulation, and improved their imaging capabilities. In contrast, only one study has been published with hydrophobic, IO NPs embedded in the lipid bilayer of liposomes (so-called B-MLs), which demonstrated superior MRI imaging capabilities, compared to free hydrophilic IO NPs [16]. This has opened the door for a new class of promising MLs as *T*_2_ contrast agents. However, more studies are warranted to assess the effect of the lipid bilayer composition on the MRI properties of B-MLs. Longitudinal (*r*_1_) or transversal relaxivity (*r*_2_) of any contrast agent is defined as the ratio of water relaxation rate constant normalized to its concentration. Contrast agents of high relaxivities can provide an equivalent contrast effect at relatively low doses, which reduce the systemic toxicity of these contrast agents. [1] Several factors influence the relaxation of water molecules in the vicinity of the magnetic centres, such as the NP’s magnetization and surface coatings [1,17]. The latter can affect the relaxation of water molecules in various forms, such as diffusion, retention, hydration, and hydrogen bonding [1,18]. The fluidity of the coating (i.e., lipid bilayer) has a significant influence on the relaxivity values since the accessibility of water protons to the magnetic centres is modified. The fluidity of the lipid bilayer depends on several factors, such as phospholipid hydrocarbon length and saturation, and cholesterol content, which has been shown to influence the MRI signals of Gd-chelates containing liposomes (*T*_1_ contrast agents) [19]. However, no such study has been carried on superparamagnetic NPs (*T*_2_ contrast agents). Although the use of an appropriate coating can boost the NP’s MRI contrast agent efficiency, this topic has been largely understudied. To the best of our knowledge, this article represents the first systematic study on the influence of different phospholipids on the *r*_2_ values of B-MLs. It was demonstrated that a lipid bilayer surface with high hydration number, which favors the diffusion of water molecules within the NPs second sphere, is crucial to obtain high *r*_2_ values. Therefore, embedding small IO NPs in the lipid bilayer leads to unprecedented improvement of *r*_2_ values compared to “free” IO NPs. Moreover, the correlation between the bilayer fluidity and relaxivity *r*_2_ is clearly shown. Finally, the in vitro MRI measurements together with the inductively coupled plasma mass spectrometry (ICP-MS) analysis revealed MLs as highly selective contrast agents that were preferentially taken up by cancerous T24 cells, which led to an improvement in the contrast and an easier distinction between the healthy and the cancerous cells at very low IO NPs concentrations, which is not the case for “free” IO NPs.

## 2. Materials and Methods

### 2.1. Chemicals

Oleic acid (OA, >99.9%), octyl ether (99%), iron (0)-pentacarbonyl (Fe(CO)_5_, > 99.99%), hydrocaffeic acid (HCA, 98%), tetrahydrofuran (THF, anhydrous, >99.9%), dimethyl sulfoxide (DMSO, > 99.5%), and cholesterol (>99%) were purchased from Sigma Aldrich (Dorset, UK). Nitric acid (65% HNO_3_), hydrochloric acid (30% HCl), and hydrogen peroxide (30% H_2_O_2_) were obtained from Merck Millipore (Milford, MA, USA). Standard solution of Fe (1000 µg Fe/mL in 2–3% HNO_3_) was obtained from Merck (Darmstadt, Germany). 1,2-dioleoyl-*sn*-glycero-3-phosphatidylcholine (DOPC), 1,2-dipalmitoyl-*sn*-glycero-3-phosphatidylcholine (DPPC), 1,2-distearoyl-*sn*-glycero-3-phophatidylcholine (DSPC), and N-[carbonyl-methoxy(polyethylene glycol)-2000]-1,2-distearoyl-*sn*-glycero-3-phosphoethanolamine, sodium salt (DSPE-PEG_2000_) were a kind gift from Lipoid GmbH (Ludwigshafen, Germany).

### 2.2. Synthesis of IO NPs

IO NPs were synthesized using thermal decomposition [20]. Oleic acid (6.08 mmol) was dissolved in 20 mL of octyl ether under a nitrogen atmosphere. The mixture was magnetically stirred and heated until the temperature reached 100 °C. After this, Fe(CO)_5_ (6.08 mmol) was injected into the mixture, and the resulting mixture was slowly heated and refluxed at 290 °C for 2 h. During reflux, the yellow-orange colour of the solutions changed to colourless then to black due to the thermal decomposition of Fe(CO)_5_ and iron oxide nanoparticle nucleation. After 2 h, the solution was cool aerated for 14 h at 80 °C, and then refluxed for 2 h (aerated iron oxide). The aerated solution was purified by adding ethanol followed by centrifugation (6000 rpm × 30 min). This process was repeated three times. Formed NPs were dispersed in CHCl_3_ and stored at 4 °C.

### 2.3. Preparation of HCA-Coated IO NPs

Hydrophilic NPs were obtained via ligand exchange reaction, where OA ligand was replaced with hydrocaffeic acid (HCA). Briefly, hydrophobic IO NPs (20 mg) were dispersed in 1 mL of tetrahydrofuran (THF) [21], and a solution of the ligand was prepared by dissolving 50 mg of HCA in 5 mL of THF. Hydrophobic NPs were added dropwise to the ligand solution, and the mixture was then stirred at 50 °C for 3 h to complete the reaction. Upon cooling the reaction mixture to room temperature, 0.5 mL of 0.5 M NaOH was added to precipitate the NPs, which were collected using centrifugation and redispersed in water and stored at 4 °C.

### 2.4. Preparation of NDPM-Coated IO NPs

Nitrodopamine palmitate (NDPM) ligand was synthesized as described in the literature [22]. NDPM-coated IO NPs were prepared by exchanging the oleic acid coating on the surface of IO NPs with NDPM ligand. Briefly, OA-IO NPs were completely dried (15 mg), then dissolved in THF (5 mL). In a separate vial, NDPM ligand (30 mg) was dissolved in a solution of DMSO/THF (1:2) (1.5 mL). OA-IO NP solution was transferred to a 15 mL Falcon tube, and the NDPM ligand solution was added dropwise. The obtained mixture was bath sonicated for 30 min and then transferred to a water bath at 50 °C for 24 h under constant magnetic stirring. The obtained NDPM-IO NPs were purified from the free ligand using centrifugation. The NP dispersion was transferred to the 15 mL Falcon tube and centrifuged for 1 h at 9000 rpm. After centrifugation, a black precipitate was formed at the bottom of the Falcon tube. The supernatant was collected, and the precipitate was washed with fresh THF (4 mL), followed by 1 h centrifugation, as described above. The washing and centrifugation steps were repeated three times. The purified NDPM-IO NPs were dissolved in chloroform (CHCl_3_) to obtain a final concentration of 5 mg/mL and stored at 4 °C.

### 2.5. Preparation of MLs

MLs were prepared using a thin-film hydration method followed by extrusion. Briefly, 0.3 mg of NDPM-IO NPs (1 mg/mL) and 10 µmol of phospholipid were dissolved in CHCl_3_. For cholesterol-containing formulations, 5 µmol of cholesterol was also included. The organic mixture was transferred to a round bottom flask (25 mL), then removed at 40 °C using a rotary evaporator (Rotavapor R-100 equipped with heating bath, Interface I-100 and vacuum pump V-100, Bunchi, Switzerland). Once the lipid film was formed, the pressure in the flask was reduced to 33 mbar for 2 h. The dried lipid film was then hydrated with 2 mL of 240 mM ammonium sulfate buffer ((NH_4_)_2_SO_4_) pH 5.4) at 60 °C for 1 h to achieve a final phospholipid concentration of 5 mM. To ensure efficient hydration, lipid-film containing flasks were briefly warmed in a circulating water bath to the desired temperature, followed by the addition of the pre-warmed buffer solution. Liposome size was reduced using the mini-extruder (Avanti Polar lipid, Alabama, USA). Samples were extruded at 60 °C through 800 nm (5 cycles) and 200 nm (15 cycles) membrane filters (PC membranes, Avanti Polar lipid, Alabama, USA). Due to the hydrophobicity, non-embedded NPs remain on the membrane filter and are therefore removed during the extrusion process. Liposomes and magneto-liposomes were left to anneal for 2 h at room temperature, then flushed with nitrogen and stored at 4 °C for further experiments.

### 2.6. Hydrodynamic Diameter and Zeta Potential Measurements

The hydrodynamic diameter, polydispersity index (PDI), and zeta potential (ζ-potential) of empty and MLs were determined using Zetasizer Nano ZS90 (Malvern, UK) at 25 °C. Disposable polystyrene cells and folded capillary cells (Malvern, UK) were used for dynamic light scattering (DLS), and zeta potential measurements, respectively. Suspensions were diluted in 0.2 μm filtered deionized water at ratios of 1:10 (v/v) for both DLS and zeta-potential measurements.

### 2.7. Fourier-Transform Infrared Spectroscopy (FTIR) Measurements

FTIR measurements were performed in order to evaluate the success of the ligand binding to the NPs’ surfaces. This was done using a Spectrum 400 spectrometer (Perkin Elmer, Waltham, MA, USA). The spectra were recorded on dried samples in the wavenumber range 4000–650 cm^−1^.

### 2.8. Magnetic Measurements

For the magnetic characterization, IO NPs were prepared in the form of a dry powder (10 mg). Magnetic measurements were performed with a vibrating-sample magnetometer (VSM MicroSense model FCM 10) at room temperature.

### 2.9. Inductively Coupled Plasma Mass Spectrometry (ICP-MS)

Total concentrations of Fe in the analysed samples were determined using an Agilent 7700 ICP-MS instrument (Agilent Technologies, Tokyo, Japan). A standard solution of Fe was diluted with water for the preparation of fresh calibration standard solutions. For the determination of Fe in aqueous suspensions of IO NPs and MLs, 0.1 mL of the initial sample was mixed with 1 mL of nitric acid (65% HNO_3_, suprapure) and heated in an oil bath at 80 °C for 1 h. After the digestion, all samples were diluted with MilliQ water to a final volume of 10 mL, and appropriately diluted prior to ICP-MS measurements. Digestion was performed in triplicates. Fe content in each sample was determined using an Fe standard curve. For the determination of the Fe concentrations in the cell samples, 2 mL of sample was digested with 0.75 mL of HCl, 0.5 mL of HNO_3_, and 1.25 mL of hydrogen peroxide (30% H_2_O_2_) and heated at 90 °C overnight with the use of a heating oven (Binder GmbH, Tuttlingen, Germany). After the digestion, the sample volume was completed with MilliQ water to a final volume of 10 mL and appropriately diluted prior to the ICP-MS measurements. All the dilutions of the samples were made with ultrapure water (18.2 MΩ cm) obtained from a Direct-Q 5 Ultrapure water system (Merck Millipore, Milford, MA, USA).

### 2.10. Transmission Electron Microscopy (TEM)

Samples for the TEM analysis were prepared by adding 5 µL of MilliQ water-diluted ML suspension on a carbon-coated 400 mesh copper grid (Ted Pella, Inc., Redding, CA, USA) and air-dried. Sample grids were examined with a JEOL JEM-1400 Plus transmission electron microscope, operating at an accelerating voltage of 120 kV.

### 2.11. Nuclear Magnetic Resonance (NMR)

^1^H NMR spectra were recorded using a Bruker Avance III 400 MHz spectrometer. 1–2 mg of each sample was dissolved in 0.5 mL of deuterated solvent. Dopamine hydrochloride, nitrodopamine, and the NDPM ligand were dissolved in deuterated DMSO (99.9 atom % D, Sigma Aldrich). NHS-palmitate ester was dissolved in 0.5 mL of deuterated chloroform (99.8 atom % D, Sigma Aldrich).

### 2.12. NMR Relaxivity Measurements

An aqueous suspension of HCA-IO NPs and MLs was prepared in a concentration range (0.25–5 µg/mL = 0.0045–0.0895 mM) with respect to the Fe content. Relaxation time measurements of HCA-IO NP and ML suspensions were carried out using an NMR/MRI system consisting of a 9.4-T superconducting magnet (Jastec, Kobe, Hyogo, Japan), and a Redstone NMR spectrometer (TecMag, Houston TX, USA). The *T*_1_ relaxation times were measured using an inversion-recovery sequence with 14 different inversion times, ranging from 50 µs to 10 s, while the *T*_2_ relaxation times were measured using the Carr Purcell Meiboom Gill (CPMG) sequence with multiple spin-echoes. The *T*_1_ and *T*_2_ relaxation times were calculated from the best fits between the measurements and the corresponding model for either *T*_2_ relaxation (exponential dependency of the echo-signal on the echo number) or *T*_1_ relaxation (dependency of the inversion recovery signal on the inversion time). The calculations were performed using the Origin program (OriginLab Corporation, Northampton MA, USA). The dependencies of the longitudinal (1/*T*_1_) and transverse (1/*T*_2_) relaxation rates on the Fe concentration in the sample were used to extract the relaxivities *R*_1_ and *R*_2_. These are defined as proportionality constants between the contrast-agent-induced increase of the corresponding relaxation rate and the MR contrast-agent concentration.

$$R_{1}=\frac{{\left(\frac{1}{T_{1}}\right)}_{C}-{\left(\frac{1}{T_{1}}\right)}_{0}}{C}\;,\;\;\;\;R_{2}=\frac{{\left(\frac{1}{T_{2}}\right)}_{C}-{\left(\frac{1}{T_{2}}\right)}_{0}}{C}$$

Here, *C* denotes the contrast-agent concentration, while the subscripts *C* and 0 denote the relaxation rates at the contrast-agent concentration *C* and zero, respectively.

Cell suspensions were centrifuged in 5-mm NMR tubes (Carl Roth, length 100 mm, outer diameter 4.95 mm, inner diameter 4.15 mm) at 1300 rpm for 5 min. The supernatant was completely removed, and an as-prepared pellet of cells was used in the relaxivity measurements. The same conditions for the *T*_1_ and *T*_2_ relaxation times were used as in the case of the suspensions. The tubes with cell suspensions were arranged in a rosette-like stack and then MRI scanned in the transverse orientation to the tubes with single- and multi-spin-echo sequences to obtain a *T*_1_-weighted and a series of differently *T*_2_-weighted images, respectively. Geometrical and resolution parameters were identical in both imaging experiments: field of view 20 mm, imaging matrix 256 by 256, no slice selection was used, while contrast parameters were TE/TR = 2.7/500 ms (echo time /repetition time) for the *T*_1_-weighted image and TE/TR = 13/2000 ms (inter-echo time/repetition time), number of echoes 8 for the series of *T*_2_-weighted images.

### 2.13. In Vitro Experiments

The experiments with urothelial cells were approved by the Veterinary Administration of the Slovenian Ministry of Agriculture and Forestry in compliance with the Animal Health Protection Act and the Instructions for Granting Permits for Animal Experimentation for Scientific Purposes. Primary cultures of normal porcine urothelial cells (NPU) cells were established from two porcine urinary bladders (biological replicates), which were obtained from a local abattoir. Human bladder muscle-invasive urothelial neoplasm (T24) cells were obtained from ATCC (LGC Standards, Germany). Primary cultures of normal porcine urothelial cells (NPU), passages IV to XII, and a cell line of human cancer urothelial cells, derived from muscle-invasive neoplasm (T24), were established as described previously [23,24,25]. Briefly, the NPU cells and T24 cells were seeded into plastic Petri dishes/6-well plastic plates (seeding density 1 × 10^5^ cells/cm^2^ and 5 × 10^4^ cells/cm^2^ for NPU and T24 cells, respectively; TPP, Trasadingen, Switzerland). The NPU cells were maintained in the culture for 4 weeks and the T24 cells for a week to establish normal and cancer urothelial models, respectively. Cells were cultured in a CO_2_-incubator at 37 °C under a humidified atmosphere of 5% CO_2_ (v/v) until they reached >85% confluency. At that point, cultures were divided into i) control groups and ii) experimental groups. Fe content in the NP suspensions was determined via ICP-MS analysis and both samples, i.e., HCA-IO NPs and MLs (DOPC/Chol/DSPE-PEG_2000_), were prepared either by dilution of HCA-IO NPs or a concentration of MLs containing 4 µg Fe/mL. The total lipid content in the case of the MLs was 1.8 mg/mL (12 mM). After the 24 h incubation, all the cells were gently washed in a fresh culture medium and were further processed for cell-viability assays and MRI analysis. After the incubation with NPs, cell-viability assays were performed using hemocytometer and trypan blue staining of dead cells and hemocytometer counting. The number of viable cells was obtained by subtracting the number of dead cells from all the counted cells. The percentage of viable cells (% Viability) in a given sample was determined as the ratio between the number of viable cells (N_S_) and the number of all the cells in the sample (N_0_): % Viability = (N_s_/N_0_) × 100%.

## 3. Results

### 3.1. Preparation and Characterization of Nitrodopamine Palmitate (NDPM)-Coated Magnetic Nanoparticles

**Figure 1:**
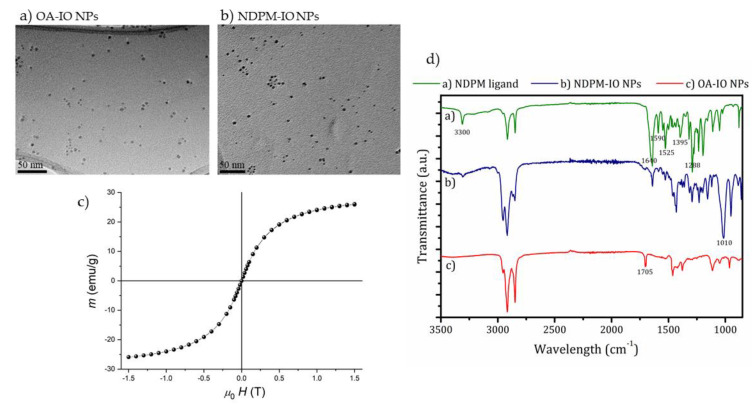
Characterization of nitrodopamine palmitate (NDPM)-coated IO NPs (iron oxide nanoparticles). Transmission Electron Microscopy (TEM) images of (**a**) Oleic acid (OA)-coated IO NPs and (**b**) NDPM-coated IO NPs; (**c**) magnetic measurements of OA-coated IO NPs at 300 K, (**d**) FTIR spectra of Fourier-Transform Infrared Spectroscopy (FTIR) spectra of the soluble NDPM ligand, NDPM-coated IO NP and OA-coated IO NPs.

Iron oxide nanoparticles (IO NPs) were synthesized using a thermal decomposition method [20]. The synthesized oleic acid (OA)-coated IO NPs were 4.5 ± 1 nm in diameter, as confirmed by the transmission electron microscopy (TEM) analysis (Figure 1a). The magnetic measurement of the OA-coated IO NPs (Figure 1c) confirmed the superparamagnetic nature of NPs with a saturation magnetization of 26 emu/g, which is a typical value for γ-Fe_2_O_3_ NPs of 4 nm nanoparticles [26]. Furthermore, we modified their surface coatings to enhance their stability and interaction with the lipid bilayer. Nitrodopamine is a versatile molecule that can be easily modified to serve as an anchor group for the functionalization of IO NPs [22]. The primary amine can act as the nucleophile; therefore, the N-hydroxy succinimide (NHS)–ester amine coupling reaction was used to synthesize nitrodopamine palmitate (NDPM) by reacting nitrodopamine with NHS palmitate according to the two-step protocol described in the literature [22]. The detailed procedure of the synthesis of the NDPM ligand is described in the Supplementary Materials, and proton nuclear magnetic resonance (^1^H NMR) spectroscopy was used to confirm the successful synthesis of NDPM. NMR spectra together with the identified peaks of the initial reagents (dopamine hydrochloride and NHS-palmitate), the intermediate product (nitrodopamine), and the final product (NDPM) are shown in Figure S1.

The OA coating on IO NPs was further replaced with NDPM, to enhance their stability and interaction with the liposome lipid bilayer (Scheme S1). A TEM image of the NDPM-coated IO NPs is shown in Figure 1b and indicates no structural changes and a good dispersion of NPs. Fourier-transform infrared (FTIR) spectroscopy was used to confirm the successful ligand-exchange reaction. FTIR spectra of the soluble NDPM ligand and NDPM-coated IO NPs are shown in Figure 1d. The FTIR spectrum obtained for the OA-coated IO NPs confirmed the presence of the adsorbed OA on the surface of the IO NPs, with a good match of the assigned bands (listed in Table S1) with that reported in the literature for similar nanoparticles [27]. Characteristic bands for the soluble NDPM ligand are listed in Table S2. A comparison of all three FTIR spectra revealed the successful exchange of the OA with the NDPM ligand on the surface of the IO NPs. The characteristic band at 1705 cm^−1^ for the C=O stretch of the hydrogen-bonded OA was not present in the FTIR spectrum for NDPM-coated IO NPs, which indicates complete ligand exchange. The following bands were present in both FTIR spectra for the pure NDPM ligand and the NDPM-coated IO NPs, which therefore indicates the presence of the NDPM ligand on the surface IO NPs: the band assigned to the asymmetric stretching of the N–H bond (*ν*_as_(N–H)) at 3300 cm^−1^, the symmetric stretching of C=O at 1640 cm^−1^, and N–H bending at 1590 cm^−1^ confirmed the presence of the amide group, which was found in the NDPM ligand. Two strong bands representing N–O stretching of the NO_2_ group at 1525 and 1395 cm^−1^ and the band corresponding to the C–N stretching at 1288 cm^−1^ were also present, but the most prominent was the strong increase in the C–O stretching at 1110 cm^−1^ in the FTIR spectra for NDPM-coated IO NPs, indicating the binding of the NDPM ligand to the NPs’ surface [28], which was further confirmed by the reduction in the intensity of the C–O stretching of the catechol group *ν*(C–OH)_aromatic_ at 1195 cm^−1^.

**Scheme 1:**
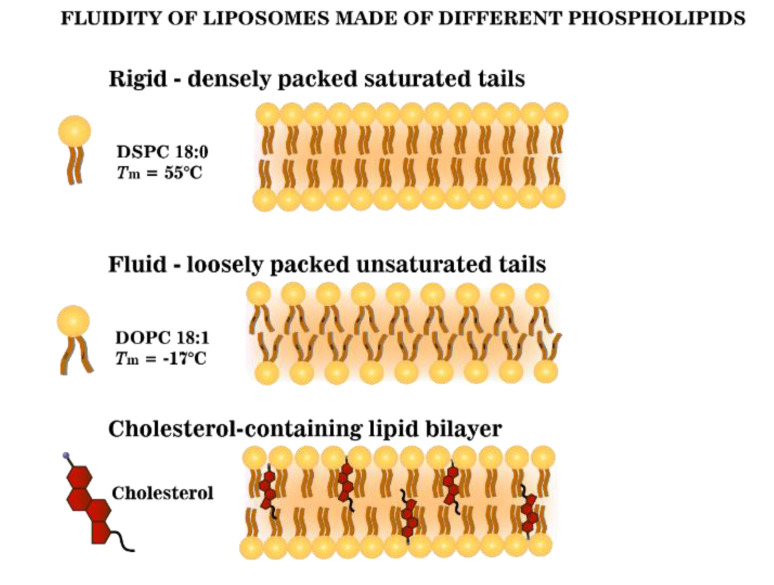
Schematic representation of the fluidity of lipid bilayers made of different phospholipids.

In our study, three different phospholipids, namely DPPC (C16:0), DSPC (C18:0), and DOPC (C18:1) were used (Figure S2). These lipids were carefully selected due to the differences in their chain length and saturation, which affect the liposomal fluidity and water accessibility (Scheme 1) [16]. The longer lipid chain of saturated lipids (DPPC and DSPC) exhibits a high phase transition temperature (*T*_m_) (41 and 55 °C, respectively). While the presence of a double bond in the C18 carbon chain/tail (DOPC (C18:1)) strongly decreases the *T*_m_ (*T*_m_ = −17 °C) (Figure S2). Cholesterol was also included in the formulations to enhance the stability and to study its influence on the bilayer fluidity (Scheme 1). Free hydrophilic hydrocaffeic acid(HCA)-coated IO NPs were used in all experiments for comparison. The physicochemical properties of all magneto-liposomes (MLs) prepared in this study are shown in Table 2. All formulations contain 4 mol % of DSPE-PEG_2000_. For the sake of clarity, hereinafter, this component will no longer be shown in the sample names used in the text.

**Figure 2:**
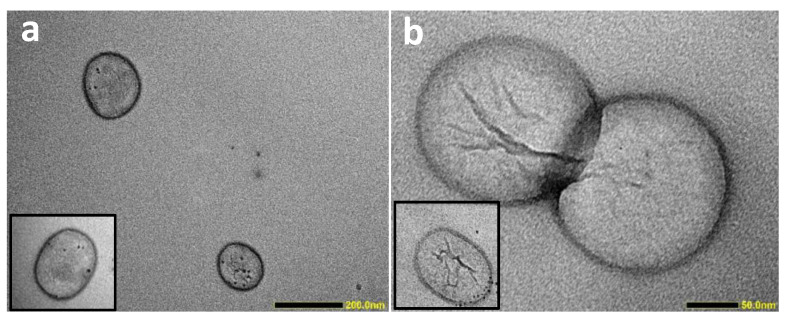
Structural elucidation of MLs. TEM images of MLs consist of (**a**) DOPC/DSPE-PEG_2000_ (96/4) and (**b**) DSPC/Chol/DSPE-PEG_2000_ (96/50/4). Scale bar is 200 nm and 50 nm, respectively.

All ML formulations have a mean hydrodynamic radius in the range 135–155 nm with a low polydispersity index (PDI) of around 0.1. The size for the cholesterol-containing formulations was slightly larger than for the non-cholesterol-containing formulations. The zeta potential results of all liposomal formulations were close to zero, as expected with PEGylated phospholipids. It is important to emphasise that good colloidal stability was observed with all our MLs (Supplementary Materials, Figure S3), since the exposure of hydrophobic NPs to the aqueous medium could result in NP aggregation and/or precipitation, which was not observed in our study. MLs of different formulation exhibited brownish colour of different intensities (Figure S3), indicating variations in NDPM-IO NP content in the sample. In order to quantitatively determine the amount of Fe in each sample, ICP-MS analysis was performed, and the obtained values are listed in Table 2. The highest Fe content was detected in the DSPC (15.90 µg/mL), followed by the DPPC (5.32 µg/mL), and DOPC (0.50 µg/mL) formulations. The Fe content results inversely correlated with the fluidity of their lipid bilayer, where the fluidity was the highest for the unsaturated DOPC and decreased by shortening of the tail length of saturated phospholipids (DPPC > DSPC). It is known that cholesterol reduces the fluidity of the bilayer [29,30]. Therefore, incorporating cholesterol into the formulation significantly lowered the Fe content of the DSPC/Chol (1.76 µg/mL) and DPPC/Chol (1.22 µg/mL) formulations, while the Fe content for DOPC/Chol remained low (0.65 µg/mL), comparable to DOPC. Finally, structural elucidation using TEM of the MLs of DOPC/Chol and DSPC/Chol revealed spherical structures of ~150 nm (Figure 2), which agreed with the DLS results. Furthermore, only small numbers of dark particles were present in the examined MLs, reflecting the low concentration of IO NPs, as determined using ICP-MS.

### 3.2. MRI Relaxivity Measurements of MLs

The effectiveness of our MLs as MRI contrast agents was investigated by measuring the dependence of the longitudinal (*T*_1_) and transverse (*T*_2_) relaxation times using a range of Fe concentrations. IO NPs from the same batch were used for the preparation of MLs and hydrophilic HCA-coated IO NPs, to reduce batch-to-batch variations. Samples were prepared with different Fe content (0.25–5 µg/mL = 0.0045–0.0895 mM), and the *T*_1_ and *T*_2_ relaxation times are presented in Figures S4 and S5 in Supplementary Materials, respectively. HCA-coated IO NPs, containing no phospholipids on the surface, were used as a control. A very important observation was the excellent stability of all the liposomal formulations after the exposure to the magnetic field. On the contrary, despite the good colloidal stability of HCA-coated IO NPs, we could observe evidence of NP agglomeration after the relaxivity measurements (data not shown). This further indicates the suitability of liposomal coating for MRI.

Magnetic NPs are considered as *T*_2_ contrast agents; therefore, all our samples had a small effect on *T*_1_ relaxation time (Supplementary Materials, Figure S4). The HCA-IO NP and DPPC/Chol *r*_1_ values were 0.4 ± 0.1 and 0.6 ± 0.1mM^−1^ s^−1^, respectively. For all other MLs, the *r*_1_ values were close to zero; therefore, it was concluded that *r*_1_ for all the samples was below 0.5 mM^−1^ s^−1^, and any further interpretation of these data would be incorrect. On the other hand, MLs have a stronger effect on the shortening of the *T_2_* relaxation time than free NPs (HCA-coated), which indicates MLs have superior MRI imaging capabilities (Supplementary Materials, Figure S5). The transversal relaxation rate increased (1/*T*_2_—1/*T*_2_(0)) as a function of Fe concentration, where *T*_2_(0) represents the *T*_2_ relaxation times of water (Supplementary Materials, Figure S6). Calculated longitudinal (*r*_1_) and transverse (*r*_2_) relaxivities of different MLs and HCA-IO NPs are summarized in Table 3.

Interestingly, all *r*_2_ values of our MLs were very high (>150 mM^−1^ s^−1^), so dividing such values with an *r*_1_ lower than 1 mM^−1^ s^−1^ would give a significantly higher *r*_2_/*r*_1_ ratio than the proposed limit of 10 [31]. In conclusion, our MLs exhibited higher *r*_2_ values than the hydrophilic HCA-IO NPs (Table 3). Among the non-cholesterol formulations, DOPC MLs showed the highest *r*_2_ relaxivity value of 673 ± 12 mM^−1^ s^−1^, followed by DPPC and DSPC MLs (283 ± 5 and 156 ± 42 mM^−1^ s^−11^, respectively). The obtained results are in good agreement with the literature [16], where the *r*_2_ relaxivity value of DOPC liposomes containing OA-coated IO NPs (*r*_2_ = 630 mM^−1^ s^−1^) was almost 2-fold the value obtained with DMPC liposomes. Upon cholesterol incorporation, the *r*_2_ values of DOPC/Chol and DPPC/Chol MLs decreased compared to the values of their non-cholesterol formulations (Table 3), as was previously reported by Carvalho et al. [11]. On the contrary, and expectedly, two batches of samples and three independent measurements showed that DSPC/Chol MLs had a higher *r*_2_ value (389 ± 9 mM^−1^ s^−1^) than the non-cholesterol containing counterpart (154 ± 4 mM^−1^ s^−1^), which requires further investigation.

### 3.3. In Vitro MRI Experiments

Following an assessment of their MRI effectiveness, DOPC/Chol MLs were selected for in vitro testing, since they demonstrated a high *r*_2_ value in the presence of cholesterol. HCA-coated IO NPs were used as a control. Both samples were incubated with normal porcine urothelial cells (NPU) and cancer urothelial cells (T24). Interestingly, our cell viability results demonstrated high biocompatibility (>90%) of both samples in normal NPU and the cancer T24 cells, following 24 h incubation (Figure S7). Next, in order to quantitatively assess the NPs’ uptake, ICP-MS analysis was used to measure Fe levels in each sample, following subtracting the endogenous Fe background (0.44–0.47 μg/mL) (Table 4). Comparable Fe content was observed in NPU and T24 cells incubated with HCA-IO NPs (0.42 and 0.44 μg/mL, respectively). This high cellular uptake (~11%) could be attributed to the small size of these HCA-IO NPs and the extended hours of incubation. On the other hand, for ML samples, higher Fe content was detected in T24 cancer cells compared to non-cancerous NPU cells (0.38 and 0.20 μg/mL, respectively), resulting in almost a two-fold increase in their cellular uptake (9.5 vs 5.0%). This observation agrees with the high nanoparticles uptake of cancer cells [32]. These interesting results highlight that MLs have higher selectivity to cancer cells, compared to small HCA-IO NPs, which could enhance MRI imaging in vivo.

**Figure 3:**
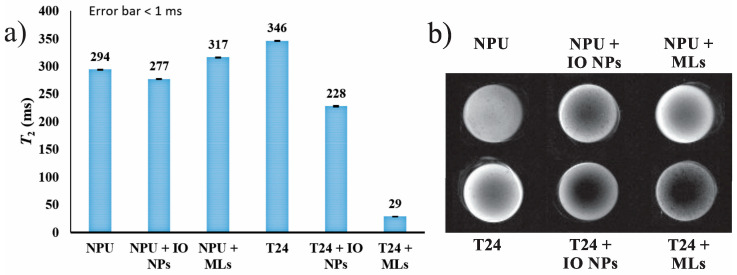
Magnetic resonance imaging (MRI) measurements of MLs in NPU and T24 cells. (**a**) T_2_ relaxation times and (**b**) T_2_-weighted image taken from cell pellets following 24 h of incubation with HCA-IO NPs or MLs (DOPC/Chol formulation). Untreated cells were used as controls. Imaging parameters were TE/TR = 13/2000 ms (inter-echo time/repetition time).

Next, in order to correlate the level of cellular uptake of the NPs and MLs with their MRI signals, the *T*_2_ relaxation times were measured for all cell pellets and are shown in Figure 3a. MRI is a very sensitive technique and can detect very small changes in the water proton environment. This is evident from the comparison of the NPU cells (294 ms) and T24 controls (346 ms), where the T24 control appears slightly brighter on the *T*_2_ image than the NPU control (Figure 3b). This is attributed to the fact that cancer cells contain more water than the normal cells [33], and therefore, a longer relaxation time for the T24 control was observed than for the NPU control. Classified as *T*_2_ contrast agents, superparamagnetic IO NPs have a strong influence on the relaxation of the water protons. Despite the fact that the concentration of IO NPs in the T24+IO NPs sample is “only” 2 µg/mL higher than in NPU+IO NPs, this difference is sufficient to observe a significant *T*_2_ decrease (*T*_2_ for T24+IO NPs and NPU+IO NPs is 228 and 277 ms, respectively) compared to the control values (Figure 3a). Furthermore, the following comparison demonstrates the importance of the relaxivity value of the added contrast agent. Despite the higher cellular uptake of HCA-IO NPs than MLs (11 and 9.5%, respectively) by cancerous T24 cells, a drastic reduction of 92% was observed in the *T*_2_ relaxation time (29 ms) in the MLs cells pellet compared to a moderate reduction of 34% in the *T*_2_ value (228 ms) of the HCA-IO NPs cells pellet. This strong reduction in *T*_2_ relaxation time was reflected in the much darker contrast of T24+MLs compared to the other samples (Figure 3b). In contrast, the lower uptake of MLs by the NPU cells resulted in a slight increase of the *T*_2_ value and brighter contrast (Figure 3a,b, respectively), probably due to the increased water content caused by the liposomal aqueous core. Overall, MLs caused a minimal change in the MRI contrast of NPU cells. However, cancer cells containing MLs appeared much darker, which is crucial for a cancer diagnosis. On the other hand, the high uptake of HCA-IO NPs in both cell lines makes the differentiation between cancer and healthy cells relatively difficult. These results revealed that MLs could improve the contrast between the healthy and the cancerous tissues at lower Fe concentration than HCA-IO NPs, proving that MLs have a high potential as promising MRI contrast agents for in vivo applications.

## 4. Discussion

IO NPs have been intensively investigated as potentially superior contrast agents for MRI when compared with the most widely used Gd-based complexes, due to the high magnetic susceptibility and lower toxicity. Numerous reports can be found about the optimal size, shape, crystallinity and composition for IO NPs to yield a high *r*_2_ value [34]. Since the proton relaxation occurs mainly at the surface or in close proximity of the NPs, the coating of the NPs plays a crucial role in the relaxation process [1]. However, this topic has been largely understudied. Thus, in the scope of this article, we attempt to correlate the NP coating properties such as fluidity and hydration number to the NPs’ performance as MRI contrast agents. The liposomal structure offers a design of a biocompatible, multifunctional system, where targeting/imaging/treatment can be done simultaneously by incorporating different components either in the liposomal core or in the bilayer. The biggest advantage of using such MLs where magnetic NPs as contrast agents are located in the bilayer is that the entire volume of the core remains available for other active components, such as drugs. Magneto-liposomes containing IO NPs in the bilayer have been investigated for magnetic hyperthermia applications [35,36,37]. However, there is only one study reporting MLs with IO NPs in the liposomal bilayer for MRI purposes [16], where OA-coated IO NPs were used for the preparation of DMPC and DOPC-based liposomes and *r*_1_ and *r*_2_ relaxivity values were determined without any in vitro or in vivo studies. More importantly, the same study reported the highest *r*_2_ value for a DOPC-PS formulation containing OA-coated IO NPs in the bilayer (PS = phosphatidylserine, *r*_2_ = 995 s^−1^ mM^−1^, while DOPC alone has *r*_2_ = 630 s^−1^ mM^−1^) [16]. Nevertheless, no explanation was given for such behaviour. PS lipid might have contributed to a negatively charged surface, thus more hydrated surface [38]; however, zeta potential measurements were not performed to confirm this claim. This highlights the necessity of conducting a systematic investigation of different liposomal formulations to prepare efficient MLs as contrast agents for MR imaging, which is the main focus of the present study, prior to conducting any in vivo studies.

In the present work, we systemically investigated the effect of lipid composition (i.e., melting point and cholesterol content) on MRI capabilities. To ensure the successful incorporation of the NPs in the liposomal bilayer, small NDPM-coated NPs were used [39]. In agreement with other reports [40], our results showed that incorporating NDPM-IO NPs into the lipid bilayer of the liposomes had minimal effects on liposome physicochemical properties (Table 2). Nevertheless, the incorporation of the IO NPs was highly dependent on the membrane fluidity, which is influenced by its melting point or the cholesterol content (Table 2). Surprisingly, increasing the membrane fluidity in non-cholesterol-containing lipid bilayers decreased the NP content (DSPC > DPPC > DOPC), which could be explained by the lower ability of the fluid bilayer to accommodate and withhold nano-sized hydrophobic NPs stably. On the other hand, we have shown that increasing the rigidity of the bilayer by incorporating cholesterol dramatically reduced the concentration of NPs, particularly in high-melting point lipid bilayers (Table 2). Previously, we observed comparable results, using the same liposomal formulations loaded with hydrophobic docetaxel drug [41]. Furthermore, a similar trend was obtained following encapsulating hydrophobic quinine into DMPC, DPPC, and DSPC lipid bilayers with an increasing cholesterol content [42]. It was shown that a high concentration of cholesterol causes limited space for drug accommodation due to the steric hindrance provided by the steroid reducing the EE, which could explain the lower concentration of the hydrophobic IO NPs in our cholesterol-containing MLs.

Ultra-small IO NPs usually have very low *r*_2_ values as a consequence of lower magnetization (Figure 1c) than the one obtained for NPs of a larger size. For example, the highest reported *r*_2_ values for small NPs (4–5 nm) are in the range from 24 to 44 s^−1^ mM^−1^ [43,44]. However, an appropriate coating can boost the contrast capability of magnetic NPs [1]. In our study, incorporation of such small NPs in the liposomal formulations led to an improvement in these *r*_2_ values ranging from 153 ± 5 s^−1^ mM^−1^ for DPPC/cholesterol/DSPE-PEG (96/50/4) up to 673 ± 12 s^−1^ mM^−1^ for DOPC/DSPE-PEG (96/4). Interestingly, all our ML formulations significantly decreased *T*_2_ values at very low Fe concentration and thus exhibited high *r*_2_ values. A similar effect was shown for MLs containing hydrophilic IO NPs in the core. Bealle et al. [15] investigated MLs composed of DPPC/DSPC (90/10 mol%) lipids and loaded with citrate-coated IO NPs. When those hydrophilic NPs (For 7- and 9-nm sized IO NPs, *r*_2_ values were 114 and 204 mM^−1^ s^−1^, respectively) were encapsulated in the liposomal core, the *r*_2_ value increased to 138 and 267 mM^−1^ s^−1^, respectively. Furthermore, our systematic investigations showed that *T*_2_ relaxation times were affected by the lipid bilayer fluidity (i.e., melting point). More precisely, higher fluidity of the bilayer (DOPC > DPPC > DSPC) led to higher relaxivity (DOPC > DPPC > DSPC). A summary of these observations is listed in Table 5. In agreement with our results, Martínez-González et al. [16] showed a similar trend whereby a saturated DOPC formulation had a significantly higher *r*_2_ value (630 s^−1^ mM^−1^) than the unsaturated DMPC MLs (340 s^−1^ mM^−1^), both containing OA-coated IO NPs in the bilayer. Unfortunately, no solid explanation for such behaviour was given.

A systematic study based on Gd-based *T*_1_ contrast agents reported that low accessibility of contrast agents to water protons (i.e., less fluid coating) resulted in lower relaxivity [19]. No such correlation was shown before for *T*_2_ contrast agents; however, we believe that the theory developed for *T*_1_ contrast agents [19] can also be applied to explain the bilayer fluidity-relaxivity correlation for MLs, which will be briefly described below. A three-sphere model describes the interaction between the water protons and the magnetic centre, and therefore, the *r*_1_ and *r*_2_ relaxivities are divided into three contributions: [1] a) *the inner sphere relaxivity* (*r*^IS^), where the hydrogen nuclei from water (or other molecules) can directly bind to the magnetic metal centre, b) in *the second sphere relaxivity* (*r*^SS^) the magnetic metal centre interacts with the long-lived hydrogen nuclei (e.g., diffusing water molecules and exchangeable protons) that are not directly bound to the metal centre and c) *the outer sphere relaxivity* (*r*^OS^) that comes from the surrounding bulk water and is constant for a specific environment (e.g., *T*_1_ and *T*_2_ times for pure water are around 4000 and 2000 ms, respectively, depending on the measurement conditions). For *T*_2_ contrast agents, the contrast mainly comes from the inhomogeneity of the generated magnetic field created by the magnetic centres, whose influence is extended to the second sphere, making the *r*^SS^ the most important contributor to the *r*_2_ value [1]. This indicates that the superparamagnetic NPs as *T*_2_ contrast agents have a longer-range influence on water protons and not only on the molecules directly bound to their surface. From this perspective, the more the water molecules diffuse into the secondary sphere of NPs, the greater the possibility of relaxing these molecules. Thus, the use of coatings that may exclude water from the vicinity of the NPs, hindering water diffusion or extremely prolonging the water residency, can cause a reduction in *r*_2_. In addition, the presence of the ligands with circulating electrons (π-system, for example, double bound in unsaturated phospholipids) will enhance *r*_2_ values [1]. More precisely, during the MRI scan, magnetic NPs create a fluctuating magnetic field, and the electrons in the surrounding atoms undergo circulations. This generates small local magnetic fields of the opposite direction, which contribute to enhanced field inhomogeneity and, consequently, higher measured relaxivity *r*_2_. Therefore, not only higher fluidity but also the presence of a π bond in the DOPC is responsible for higher *r*_2_ values compared to DPPC- and DSPC-based formulations.

Furthermore, a critical factor for *r*_2_ is the diffusion dynamics of water molecules in the magnetic field gradients. This contribution is measured by the number of water molecules diffused into the secondary sphere of the contrast agent and their residency time within the region. Liposomes increase MR contrast agent hydration number and decrease the water diffusion rate caused by a phospholipid bilayer [45]. Moreover, the phosphocholine head group present in our MLs has a high hydration number, where 25–30 water molecules are needed to completely hydrate the phosphocholine head group, in contrast to the ethanolamine head group hydration number (10–12 water molecules) [46]. This indicates that more water molecules are present in the vicinity of the magnetic centres, which amplifies the MR signals. In addition, PEG molecules are responsible for a fixed aqueous layer thickness (FALT) near the liposomes, thus assisting water diffusion through the bilayer and maintaining a high hydration number [47]. Therefore, besides the stealth effect, we believe that PEGylated phospholipids, such as DSPE-PEG_2000_, play an important role in enhancing the liposomal hydration state. Furthermore, not only hydration numbers but also water exchange rates differ over three orders of magnitude when comparing different functional groups (phosphonate ~ phenolate > α-substituted acetate > acetate > hydroxamate ~ sulfonamide > amide ~ pyridyl ~ imidazole) [48], with the phosphonate group having the highest water exchange rate. These parameters could explain the MR superiority of our MLs, regardless of their lipid composition, compared to free HCA-IO NPs.

Cholesterol reduces the fluidity of the membrane and thus the *r*_2_ relaxivity value. Therefore, DOPC/Chol and DPPC/Chol formulations have lower *r*_2_ values (575 and 153 mM^−1^ s^−1^, respectively) than their non-cholesterol counterparts (673 and 283 mM^−1^ s^−1^, respectively). Martínez-González et al. [16] showed a similar effect where DOPC/Chol and DMPC/Chol liposomes containing OA-coated IO NPs exhibited lower *r*_2_ values (281 and 230 s^−1^ mM^−1^, respectively) than the non-cholesterol DOPC and DMPC formulations (630 and 340 s^−1^ mM^−1^, respectively). Unexpectedly, our MLs composed of DSPC/Chol/DSPE-PEG (63/33/4) showed a higher *r*_2_ relaxivity compared to DSPC/DSPE-PEG (96/4) MLs. Skouras et al. [49] investigated similar formulations, i.e., DSPC/DSPE-PEG (80/20) and DSPC/Chol/DSPE-PEG (76/20/4), and the relaxivity was lower for the cholesterol-containing sample (46 s^−1^ mM^−1^) than for the non-cholesterol counterpart (154 s^−1^ mM^−1^). The important differences are the lower cholesterol content, and their MLs contained hydrophilic NPs in the core, while our hydrophobic NPs are located in the bilayer, thus the water accessibility might be different. Below *T*_m_, the presence of cholesterol prevents ordered packing of lipids, thus increasing their membrane fluidity [29,30]. This could mean that the lipid bilayer of DSPC/Chol MLs is more fluid or contains more defects at room temperature than the DSPC MLs, and consequently, a higher *r*_2_ is observed. However, further investigations are still warranted to understand the behaviour of these highly stable and clinically-relevant liposomes.

Interestingly, for all the reports investigating H-MLs [11,12,13,14,15,49,50,51] (IO NPs in the core) or B-MLs [16,37] (IO NPs in the bilayer) for MRI applications, no in vitro MRI studies could be found. A detailed list of the above-mentioned references with a summary of their experimental conditions (size of IO NPs and their location in the liposomes, liposomal formulation, concentration range used for the determination of *r*_2_, maximum *r*_2_, and in vivo conditions where applicable) can be found in the Table S3 in Supplementary Materials. Promisingly, our in vitro MRI results revealed, for the first time, a clear distinction between normal and tumor cells as a consequence of different internalization of MLs and small HCA-IO NPs. It has been shown that the internalization rate strongly depends on the NPs’ size, shape, and surface properties, as well as the type of the cell line [52]. In the present study, normal cells (NPU) internalized 10.5% and 5% of initial HCA-IO NPs and MLs, respectively, while cancerous T24 cells internalized more, i.e., 11% and 9.5%, respectively. It is believed that normal cells are less likely to internalize NPs larger than 10 nm, while large NPs (>100 nm) can be easily taken up by cancerous cells, as demonstrated in our previous study [32]. Therefore, due to the very small size of the HCA-IO NPs (approx. 4.5 nm), the internalization rate for NPU and T24 cells was similar (10.5% and 11%, Table 4), while larger MLs were internalized in the NPU cells to a lesser extent (5%) than in cancerous T24 cells (9.5%). *T*_2_ measurements of the cell pellets (Figure 3) demonstrated that not only the concentration but the relaxivity of the contrast agent and the type of cell (caner vs normal) were also crucial factors for the MRI contrast enhancement. For instance, although the higher Fe content HCA-IO NPs caused a smaller reduction in *T*_2_ relaxation times in the cell pellet (T24+IO NPs was shortened only by 34%), in the case of the T24+MLs the *T*_2_ relaxation time decreased by 92% (29 ms) regarding the control sample. This can be attributed to the higher *r*_2_ relaxivity of the MLs. Furthermore, similar cellular uptake levels of IO NPs in NPU and cancerous T24 cells makes a clear differentiation between the healthy and the cancerous cells rather difficult. On the contrary, due to the low cellular uptake the MLs showed no decrease in *T*_2_ relaxation time compared to the NPU control, but even a slight increase in *T*_2_ value, which can be seen as a brighter *T*_2_ image than the NPU control. In the case of the MLs, two competitive processes have to be considered. The first is the influence of the IO NPs that decrease the *T*_2_ signal, and the second one is the influence of the water content increase coming from the liposomal aqueous core, causing the increase in the *T*_2_ signal. At low MLs cellular uptake (low Fe content), the water content increase will be a dominant contribution to the total *T*_2_ signal. Therefore, the *T*_2_ value slightly increased (NPU+MLs sample), which could be seen as a brighter *T*_2_ image (Figure 3b). On the other hand, a high uptake of MLs and consequently a high concentration of Fe in the sample (T24+MLs) shifts these two competitive processes in favor of an IO-based *T*_2_ decrease, although the water content increased and caused a strong dark contrast on the *T*_2_ image. This is an interesting phenomenon, which has not been studied in detail yet and requires a separate systematic study that involves the incorporation of different concentrations of empty and magnetic liposomes in the different types of cells.

Our results clearly demonstrate the importance of tailoring the surface properties of magnetic NPs to enhance their MR imaging capabilities for in vitro and in vivo applications. Taking into account the negligible effect of MLs on the *T*_2_ of the normal cells and a massive signal decrease in the cancerous cells, not only did MLs prove to be highly efficient, but the selectivity between the normal (non-cancerous) and cancerous cells was clearly demonstrated.

## 5. Conclusions

To the best of our knowledge, this article represents the first systematic study of the influence of different phospholipids on the *r*_2_ values of MLs containing magnetic NPs in the bilayer. Our results showed that *r*_2_ values were highly influenced by the lipid bilayer composition, which was contributed to the differences in the fluidity and the diffusion dynamics of the water molecules in the secondary sphere of IO NPs. Overall, all the prepared liposomal formulations exhibited higher *r*_2_ values than the hydrophilic HCA-IO NPs. Due to the high bilayer fluidity of DOPC-based formulations, their MRI performance was superior to other liposomal formulations. Our in vitro experiments showed that our DOPC/Chol MLs were proven to be non-toxic for normal (NPU) and cancerous (T24) cells after incubation for 24 h. Moreover, the in vitro MRI measurements revealed that MLs could serve as an excellent contrast agent, enabling an easier distinction between the healthy and the cancerous tissues, proving that the studied magneto-liposomes have a high potential to be used as an MRI contrast agent even at very low concentration. Furthermore, MLs could encapsulate a wide range of therapeutics and diagnostics in their aqueous core, offering a great platform for the preparation of multifunctional nanoparticles for theranostics and image-guided drug delivery.

## Figures and Tables

**Table 1:** List of iron oxide (IO)-based contrast agents that have been clinically approved or entered clinical trials for magnetic resonance (MR) imaging (adapted from [6,8,9,10]). Relaxivities were measured at 1.5 T.

Commercial Name	NP Size, Coating and Relaxivities (mM^−1^ s^−1^)	Intended Use, Status on the Marked
Endorem in EU/Feridex^®^ IV in USA	150 nm, dextran-coated IO NPs *r*_1_ = 10.1 and *r*_2_ = 120	Primarily designed for liver imaging, approved, and withdrawn from the market due to a lack of users.
Resovist^®^	60 nm, carbodextran-coated IO NPs *r*_1_ = 9.7 and *r*_2_ = 189	Primarily designed for liver imaging, approved, currently available only in Japan.
Gastromark^®^ in EU/Lumirem in USA	>300 nm, siloxane-coated IO NPs Relaxivity n.a.	Approved as an oral contrast agent, withdrawn from the market due to a lack of users.
Rienso in EU/Faraheme^®^ in USA	30 nm, carboxyhydrate-coated IO NPs, *r*_1_ = 15 and *r*_2_ = 89	Approved for iron-deficiency treatment, withdrawn from EU market, available in US to treat iron-deficiency anaemia in adults with chronic kidney disease, off-label use as MRI contrast agent.
Sinerem^®^ in EU/Combidex^®^ in USA	30 nm, dextran-coated IO NPs *r*_1_ = 9.9 and *r*_2_ = 65	Intended for diagnostic use in the characterisation of lymph nodes visualised by MRI, Phase III completed, application was withdrawn in 2007 before approval, failed to demonstrate a statistically significant benefit for sensitivity and specificity.
VSOP C184	Citrate coated 4-8 nm IO NPs, *r*_1_ = 14 and *r*_2_ = 33.4	Clinical trials for MR angiography, not approved.
Abdoscan^®^	3.5 µm Relaxivity n.a.	Oral gastrointestinal imaging, approved in EU but taken off the market in 2000.
Siena Plus^®^	59 nm IO NPs Relaxivity n.a.	Injected subcutaneously to detect lymph nodes with Sentimag^®^ device, approved in EU. In USA, it is limited to investigational use only.

**Table 2:** List of formulations used for the preparation of magneto-liposomes (MLs) with corresponding physicochemical properties. Hydrodynamic size, polydispersity index (PDI) and zeta potential as determined using dynamic light scattering (DLS). Fe content in MLs as measured using inductively coupled plasma mass spectrometry (ICP-MS).

Lipid Formulation	Size (nm)	PDI	ζ-Potential (mV)	Fe (µg/mL)
DOPC/DSPE-PEG_2000_ (96/4)	144 ± 3	0.09 ± 0.01	−0.6 ± 0.2	0.50 ± 0.03
DPPC/DSPE-PEG_2000_ (96/4)	150 ± 4	0.10 ± 0.04	0.8 ± 0.3	5.32 ± 0.08
DSPC/DSPE-PEG_2000_ (96/4)	135 ± 2	0.11± 0.05	−0.4 ± 0.2	15.90 ± 0.10
DOPC/Chol/DSPE-PEG_2000_ (96/50/4)	156 ± 4	0.10 ± 0.02	−0.5 ± 0.2	0.65 ± 0.05
DPPC/Chol/DSPE-PEG_2000_ (96/50/4)	155 ± 6	0.11 ± 0.03	1.9 ± 0.2	1.22 ± 0.08
DSPC/Chol/DSPE-PEG_2000_ (96/50/4)	141 ± 5	0.07 ± 0.01	0.3 ± 0.1	1.76 ± 0.08

**Table 3:** MLs as a promising MRI *T*_2_ contrast agent. Calculated longitudinal (*r*_1_) and transverse (*r*_2_) relaxivities of different MLs and HCA-IO NPs.

Sample	*r_1_* (mM^−1^ s^−1^)	*r*_2_ (mM^−1^ s^−1^)
DOPC/DSPE-PEG_2000_ (96/4)	<0.5	673 ± 12
DPPC/DSPE-PEG_2000_ (96/4)	<0.5	283 ± 9
DSPC/DSPE-PEG_2000_ (96/4)	<0.5	156 ± 4
DOPC/Chol/DSPE-PEG_2000_ (96/50/4)	<0.5	575 ± 5
DPPC/Chol/DSPE-PEG_2000_ (96/50/4)	0.6 ± 0.1	153 ± 5
DSPC/Chol/DSPE-PEG_2000_ (96/50/4)	<0.5	389 ± 9
HCA-IO NPs	0.4 ± 0.1	16 ± 3

**Table 4:** After 24 h incubation of normal (NPU) and cancerous T24 cells, Fe content in the cells was determined using ICP-MS analysis. For the calculation of cellular uptake, the endogenous Fe (control groups) was subtracted (marked as Corrected Fe).

Sample	Total Fe (µg/mL)	Corrected Fe (µg/mL)	Cellular Uptake (%)
NPU control	0.44 ± 0.06	-	-
NPU + HCA-IO NPs	0.86 ± 0.05	0.42 ± 0.06	10.5 ± 1.2%
NPU + MLs	0.64 ± 0.05	0.20 ± 0.06	5.0 ± 0.6%
T24 control	0.47 ± 0.05	-	-
T24 + HCA-IO NPs	0.91 ± 0.04	0.44 ± 0.05	11.0 ± 1.1%
T24 + MLs	0.84 ± 0.05	0.38 ± 0.05	9.5 ± 0.8%

**Table 5:** Summary of results showing the influence of the phospholipid composition of MLs on the bilayer fluidity and the *r*_2_ relaxivity values.

	Non-Cholesterol MLs Formulations	Cholesterol-Containing MLs Formulations
**Fluidity**	DOPC > DPPC > DSPC	DOPC/Chol > DSPC/Chol > DPPC/Chol
***r*_2_**	DOPC > DPPC > DSPC	DOPC/Chol > DSPC/Chol > DPPC/Chol

## Supplementary Materials

The following are available online at https://www.mdpi.com/2079-4991/10/5/889/s1, Synthesis details and NMR characterization of NDPM ligand. Figure S1: NMR spectra for reagents and the final product (NDPM ligand), Scheme S1: Schematic representation of the ligand exchange reaction, Table S1: FTIR assignment of OA-coated IO NPs, Table S2: FTIR assignment of pure NDPM ligand, Figure S2: Structure, chemical name and phase transition temperature of used lipids in this study, Figure S3: Photo of MLs suspensions with different lipids composition, Figure S4: *T*_1_ relaxation times of MLs and HCA-coated IO NPs, Figure S5: *T*_2_ relaxation times of MLs and HCA-coated IO NPs, Figure S6: Graph showing inverse transverse 1/*T*_2_—1/*T*_2_(0) relaxation rate increase for MLs and HCA-coated IO NPs, Figure S7: Cell viabilities after incubation of normal and cancerous T24 cells with HCA-IO NPs or MLs for 24, Table S3: A review table containing all published studies using MLs for MRI applications with the summary of their experimental data.

## References

[1] Zhang W., Liu L., Chen H., Hu K., Delahunty I., Gao S., Xie J. (2018). Surface impact on nanoparticle-based magnetic resonance imaging contrast agents. Theranostics.

[2] Boros E., Gale E.M., Caravan P. (2015). MR imaging probes: Design and applications. Dalt. Trans..

[3] Wang Y.-X.J. (2011). Superparamagnetic iron oxide based MRI contrast agents: Current status of clinical application. Quant. Imaging Med. Surg..

[4] Estelrich J., Sanchez-Martin M.J., Busquets M.A. (2015). Nanoparticles in magnetic resonance imaging: From simple to dual contrast agents. Int. J. Nanomed..

[5] Jin R., Lin B., Li D., Ai H. (2014). Superparamagnetic iron oxide nanoparticles for MR imaging and therapy: Design considerations and clinical applications. Curr. Opin. Pharmacol..

[6] Dadfar S.M., Roemhild K., Drude N.I., von Stillfried S., Knüchel R., Kiessling F., Lammers T. (2019). Iron oxide nanoparticles: Diagnostic, therapeutic and theranostic applications. Adv. Drug Deliv. Rev..

[7] Kneidl B., Peller M., Winter G., Lindner L.H., Hossann M. (2014). Thermosensitive liposomal drug delivery systems: State of the art review. Int. J. Nanomed..

[8] Corot C., Robert P., Idée J.M., Port M. (2006). Recent advances in iron oxide nanocrystal technology for medical imaging. Adv. Drug Deliv. Rev..

[9] Wáng Y.X.J., Idée J.M. (2017). A comprehensive literatures update of clinical researches of superparamagnetic resonance iron oxide nanoparticles for magnetic resonance imaging. Quant. Imaging Med. Surg..

[10] Pita R., Ehmann F., Papaluca M. (2016). Nanomedicines in the EU—Regulatory Overview. AAPS J..

[11] Carvalho A., Goncalves M.C., Martins M.B.F., Meixedo D., Feio G. (2013). Relaxivities of magnetoliposomes: The effect of cholesterol. Magn. Reson. Imaging.

[12] Marie H., Lemaire L., Franconi F., Lajnef S., Frapart Y.-M., Nicolas V., Frebourg G., Trichet M., Menager C., Lesieur S. (2015). Superparamagnetic Liposomes for MRI Monitoring and External Magnetic Field-Induced Selective Targeting of Malignant Brain Tumors. Adv. Funct. Mater..

[13] Garnier B., Tan S., Miraux S., Bled E., Brisson A.R. (2012). Optimized synthesis of 100 nm diameter magnetoliposomes with high content of maghemite particles and high MRI effect. Contrast Media Mol. Imaging.

[14] Faria M.R., Cruz M.M., Gonçalves M.C., Carvalho A., Feio G., Martins M.B.F. (2013). Synthesis and characterization of magnetoliposomes for MRI contrast enhancement. Int. J. Pharm..

[15] Béalle G., Di Corato R., Kolosnjaj-Tabi J., Dupuis V., Clément O., Gazeau F., Wilhelm C., Ménager C. (2012). Ultra magnetic liposomes for MR imaging, targeting, and hyperthermia. Langmuir.

[16] Martínez-González R., Estelrich J., Busquets M.A. (2016). Liposomes Loaded with Hydrophobic Iron Oxide Nanoparticles: Suitable T₂ Contrast Agents for MRI. Int. J. Mol. Sci..

[17] Lin Y., Wang S., Zhang Y., Gao J., Hong L., Wang X., Wu W., Jiang X. (2015). Ultra-high relaxivity iron oxide nanoparticles confined in polymer nanospheres for tumor MR imaging. J. Mater. Chem. B.

[18] Huang J., Zhong X., Wang L., Yang L., Mao H. (2012). Improving the magnetic resonance imaging contrast and detection methods with engineered magnetic nanoparticles. Theranostics.

[19] Ferrauto G., Delli Castelli D., Di Gregorio E., Terreno E., Aime S. (2016). LipoCEST and cellCEST imaging agents: Opportunities and challenges. WIREs Nanomed. Nanobiotechnol..

[20] Sun S., Zeng H., Robinson D.B., Raoux S., Rice P.M., Wang S.X., Li G. (2004). Monodisperse MFe2O4 (M = Fe, Co, Mn) nanoparticles. J. Am. Chem. Soc..

[21] Kostevšek N., Hudoklin S., Kreft M.E., Serša I., Sepe A., Jagličić Z., Vidmar J., Ščančar J., Šturm S., Kobe S. (2018). Magnetic interactions and: In vitro study of biocompatible hydrocaffeic acid-stabilized Fe-Pt clusters as MRI contrast agents. RSC Adv..

[22] Niebel T.P., Heiligtag F.J., Kind J., Zanini M., Lauria A., Niederberger M., Studart A.R. (2014). Multifunctional microparticles with uniform magnetic coatings and tunable surface chemistry. RSC Adv..

[23] Višnjar T., Kocbek P., Kreft M.E. (2012). Hyperplasia as a mechanism for rapid resealing urothelial injuries and maintaining high transepithelial resistance. Histochem. Cell Biol..

[24] Resnik N., Repnik U., Kreft M.E., Sepčić K., Maček P., Turk B., Veranič P. (2015). Highly Selective Anti-Cancer Activity of Cholesterol-Interacting Agents Methyl-β-Cyclodextrin and Ostreolysin A/Pleurotolysin B Protein Complex on Urothelial Cancer Cells. PLoS ONE.

[25] Kreft M.E., Hudoklin S., Sterle M. (2005). Establishment and characterization of primary and subsequent subcultures of normal mouse urothelial cells. Folia Biol. (Praha).

[26] Morales M.P., Veintemillas-Verdaguer S., Serna C.J. (1999). Magnetic properties of uniform g-Fe2O3 nanoparticles smaller than 5 nm prepared by laser pyrolysis. J. Mater. Res..

[27] Bixner O., Lassenberger A., Baurecht D., Reimhult E. (2015). Complete Exchange of the Hydrophobic Dispersant Shell on Monodisperse Superparamagnetic Iron Oxide Nanoparticles. Langmuir.

[28] Smolensky E.D., Park H.Y.E., Berquó T.S., Pierre V.C. (2011). Surface functionalization of magnetic iron oxide nanoparticles for MRI applications - effect of anchoring group and ligand exchange protocol. Contrast Media Mol. Imaging.

[29] de Meyer F., Smit B. (2009). Effect of cholesterol on the structure of a phospholipid bilayer. Proc. Natl. Acad. Sci. USA.

[30] Redondo-Morata L., Giannotti M.I., Sanz F. (2012). Influence of cholesterol on the phase transition of lipid bilayers: A temperature-controlled force spectroscopy study. Langmuir.

[31] Caravan P., Ellison J.J., McMurry T.J., Lauffer R.B. (1999). Gadolinium(III) Chelates as MRI Contrast Agents: Structure, Dynamics, and Applications. Chem. Rev..

[32] Kostevšek N., Abramovič I., Hudoklin S., Kreft M.E., Serša I., Sepe A., Vidmar J., Šturm S., Spreitzer M., Ščančar J. (2018). Hybrid FePt/SiO2/Au nanoparticles as a theranostic tool: In vitro photo-thermal treatment and MRI imaging. Nanoscale.

[33] Ross K.F.A., Gordon R.E. (1982). Water in malignant tissue, measured by cell refractometry and nuclear magnetic resonance. J. Microsc..

[34] Zhou Z., Yang L., Gao J., Chen X. (2019). Structure–Relaxivity Relationships of Magnetic Nanoparticles for Magnetic Resonance Imaging. Adv. Mater..

[35] Salvatore A., Montis C., Berti D., Baglioni P. (2016). Multifunctional Magnetoliposomes for Sequential Controlled Release. ACS Nano.

[36] Chen Y., Bose A., Bothun G.D. (2010). Controlled release from bilayer-decorated magnetoliposomes via electromagnetic heating. ACS Nano.

[37] Guo Y., Zhang Y., Ma J., Li Q., Li Y., Zhou X., Zhao D., Song H., Chen Q., Zhu X. (2018). Light/magnetic hyperthermia triggered drug released from multi-functional thermo-sensitive magnetoliposomes for precise cancer synergetic theranostics. J. Control. Release.

[38] Scheu R., Rankin B.M., Chen Y., Jena K.C., Ben-Amotz D., Roke S. (2014). Charge asymmetry at aqueous hydrophobic interfaces and hydration shells. Angew. Chem.-Int. Ed..

[39] Amstad E., Gillich T., Bilecka I., Textor M., Reimhult E. (2009). Ultrastable Iron Oxide Nanoparticle Colloidal Suspensions Using Dispersants with Catechol-Derived Anchor Groups. Nano Lett..

[40] Amstad E., Kohlbrecher J., Müller E., Schweizer T., Textor M., Reimhult E. (2011). Triggered release from liposomes through magnetic actuation of iron oxide nanoparticle containing membranes. Nano Lett..

[41] Pereira S., Egbu R., Jannati G., Al-Jamal W.T. (2016). Docetaxel-loaded liposomes: The effect of lipid composition and purification on drug encapsulation and in vitro toxicity. Int. J. Pharm..

[42] Briuglia M.L., Rotella C., McFarlane A., Lamprou D.A. (2015). Influence of cholesterol on liposome stability and on in vitro drug release. Drug Deliv. Transl. Res..

[43] Li J., Shi X., Shen M. (2014). Hydrothermal Synthesis and Functionalization of Iron Oxide Nanoparticles for MR Imaging Applications. Part. Part. Syst. Charact..

[44] Li Z., Yi P.W., Sun Q., Lei H., Li Zhao H., Zhu Z.H., Smith S.C., Lan M.B., Lu G.Q. (2012). Ultrasmall water-soluble and biocompatible magnetic iron oxide nanoparticles as positive and negative dual contrast agents. Adv. Funct. Mater..

[45] Jacques V., Dumas S., Sun W.-C., Troughton J., Greenfield M.T., Caravan P. (2010). High relaxivity MRI contrast agents part 2: Optimization of inner- and second-sphere relaxivity. Invest. Radiol..

[46] Damodaran K.V., Merz K.M., Gaber B.P. (1992). Structure and Dynamics of the Dilauroylphosphatidylethanolamine Lipid Bilayer. Biochemistry.

[47] Carvalho A., Martins M.B.F., Corvo M.L., Feio G. (2014). Enhanced contrast efficiency in MRI by PEGylated magnetoliposomes loaded with PEGylated SPION: Effect of SPION coating and micro-environment. Mater. Sci. Eng. C.

[48] Dumas S., Jacques V., Sun W.-C., Troughton J.S., Welch J.T., Chasse J.M., Schmitt-Willich H., Caravan P. (2010). High relaxivity magnetic resonance imaging contrast agents. Part 1. Impact of single donor atom substitution on relaxivity of serum albumin-bound gadolinium complexes. Invest. Radiol..

[49] Skouras A., Mourtas S., Markoutsa E., De Goltstein M.-C., Wallon C., Catoen S., Antimisiaris S.G. (2011). Magnetoliposomes with high USPIO entrapping efficiency, stability and magnetic properties. Nanomed. Nanotechnol. Biol. Med..

[50] Shen S., Huang D., Cao J., Chen Y., Zhang X., Guo S., Ma W., Qi X., Ge Y., Wu L. (2019). Magnetic liposomes for light-sensitive drug delivery and combined photothermal–chemotherapy of tumors. J. Mater. Chem. B.

[51] Martina M.S., Fortin J.P., Ménager C., Clément O., Barratt G., Grabielle-Madelmont C., Gazeau F., Cabuil V., Lesieur S. (2005). Generation of superparamagnetic liposomes revealed as highly efficient MRI contrast agents for in vivo imaging. J. Am. Chem. Soc..

[52] Kang H., Mintri S., Menon A.V., Lee H.Y., Choi H.S., Kim J. (2015). Pharmacokinetics, pharmacodynamics and toxicology of theranostic nanoparticles. Nanoscale.

