# Supplementary material for: Magneto-Liposomes as MRI Contrast Agents: A Systematic Study of Different Liposomal Formulations
Source: Nanomaterials (Basel). 2020 May 6;10(5):889. doi: 10.3390/nano10050889 (PMC7279489; doi:10.3390/nano10050889)
Supplement: Supplementary file 1 [file nanomaterials-10-00889-s001.pdf]

nanomaterials

MDPI

Supporting Information

Magneto-Liposomes as MRI Contrast Agents: A Systematic Study of Different Liposomal Formulations

Nina Kostevšek [1,*], Calvin C.L. Cheung [2], Igor Serša [3], Mateja Erdani Kreft [4], Ilaria Monaco [5], Mauro Comes Franchini [5], Janja Vidmar [6] and Wafa T. Al-Jamal [2,*]

1 Department for Nanostructured Materials, Jožef Stefan Institute, 1000 Ljubljana, Slovenia
2 School of Pharmacy, Queen's University Belfast, BT9 7BL Belfast, UK; ccheung04@qub.ac.uk
3 Condensed Matter Physics Department, Jožef Stefan Institute, 1000 Ljubljana, Slovenia; igor.sersa@ijs.si
4 Faculty of Medicine, Institute of Cell Biology, University of Ljubljana, 1000 Ljubljana, Slovenia; mateja.erdani@mf.uni-lj.si
5 Department of Industrial Chemistry "Toso Montanari", University of Bologna, 40136 Bologna, Italy; ilaria.monaco@bio-on.it (I.M.); mauro.comesfranchini@unibo.it (M.C.F.)
6 Department for Environmental Sciences, Jožef Stefan Institute, 1000 Ljubljana, Slovenia; janja.vidmar@ijs.si
* Correspondence: nina.kostevsek@ijs.si (N.K.); w.al-jamal@qub.ac.uk (W.T.A.)

Synthesis of and characterization of nitrodopamine palmitoyl (NDPM) ligand

The synthesis of nitrodopamine palmitate consisted of two steps.[1] In the first step nitrodopamine was prepared and in the second one nitrodopamine palmitate was formed.

Synthesis of nitrodopamine (I step)

1.893 g (10.0 mmol) of 3,4-hydroxytyramine hydrochloride (>98%, Sigma Aldrich) and 1.515 g (22 mmol) of sodium nitrite (>98%, Sigma Aldrich) were dissolved in DI water (26 mL) and the 100 mL flask was placed in an ice bath. A diluted solution of sulphuric acid (0.927 mL of 98% H_2SO_4 in 10 mL of DI water) was slowly added to the mixture, where a yellow precipitate was formed. After the acid addition, the ice bath was removed and the mixture was left stirring overnight at room temperature. The formed precipitate was filtered and recrystallized from water. The product was dried under a high vacuum to obtain the nitrodopamine as the hemisulfate salt (1.21 g, 6.4 mmol, yield 64 %).

Synthesis of nitrodopamine palmitate (NDPM) (Step II)

The obtained nitrodopamine as hemisulfate salt was dissolved in (2.35 mmol, 1.1 eq) in 30 mL of anhydrous DMF (99.8%, Sigma Aldrich). Then triethylamine (0.6 mL, 4.32 mmol, 2 eq) (>99 %, Sigma Aldrich) was added dropwise and the obtained solution was cooled down in an ice bath. In a separate flask, N-hydroxy succinimide palmitoyl ester (>98%, Sigma Aldrich) was dissolved (0.75 g, 2.12 mmol, 1 eq) in 20 mL anhydrous DMF. This solution was added dropwise to the nitrodopamine mixture. After that, the ice bath was removed and the reaction mixture was left overnight under magnetic stirring. To purify the product, acidified water (HCl 1%, 150 mL) was added to the reaction mixture and the obtained solution was extracted with ethyl acetate (3x100 mL, anhydrous, 99.8%, Sigma Aldrich). The combined organic phases were washed with brine (15 mL) and with DI water (150 mL). The remained water in the organic phase was removed by using anhydrous magnesium sulphate (99.99%, Sigma Aldrich). The organic solvent was concentrated to a volume of ~100 mL using a rotary evaporator. The product was precipitated as a yellow powder by cooling in a refrigerator overnight, then collected by filtration. The precipitate was washed with cold ethyl acetate and dried in an evacuated desiccator over silica gel (NDPM, 0.70 g, 1.61 mmol, yield 75.8%).

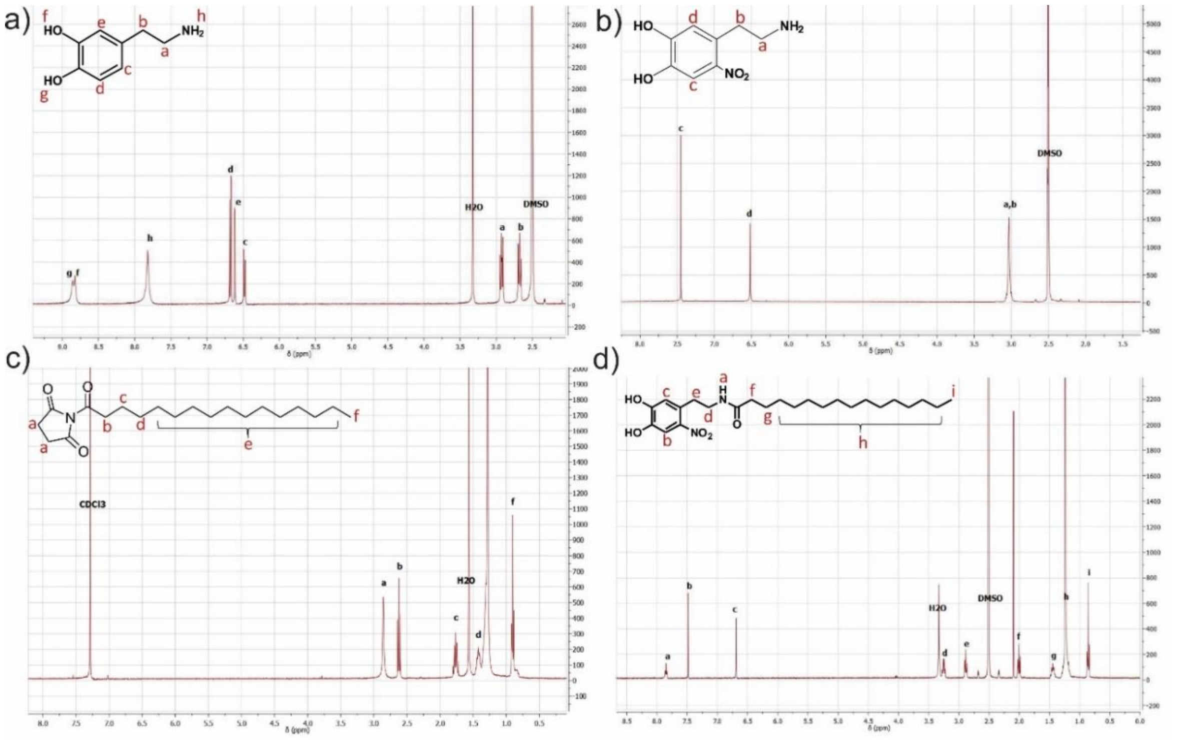

Figure S1. NMR spectra with identified peaks of the initial reagents a) dopamine hydrochloride and b) NHS-palmitate), c) the intermediate product (nitrodopamine) and d) the final product (NDPM ligand).

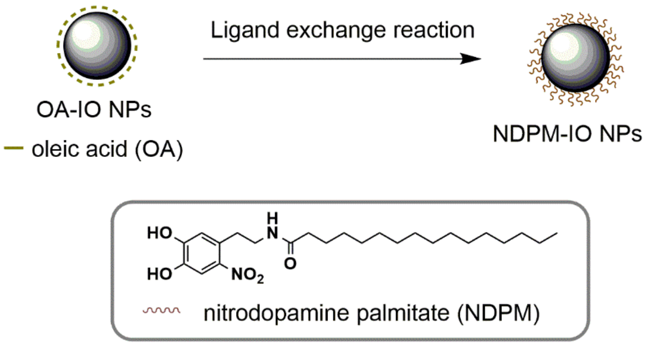

Scheme S1. Schematic representation of the ligand exchange reaction that occurred on oleic acid-coated IO NPs surface to obtain the NDPM-coated IO NPs.

Table S1. FTIR assignment of OA-coated IO NPs. [2]**.**

**Band**	**Wavenumber / cm^{-1} (intensity)**
ν_{as} (CH_3)	2956 (m)
ν_{as} (CH_2)	2917 (s)
ν_{s} (CH_3)	2872 (m)
ν_{s} (CH_2)	2849 (s)
ν_{s} (**C=O**)O-H$_{dimer}$	1705 (s)
ν_{as} (COO^-) bident	1525 (s)
δ (CH_2)	1463 (s)
δ$_{in\ plane}$ (C=O)**O-H**	1430 (m)
ν_{s} (COO^-)	1420 (m, broad)
δ (CH_3)	1380 (s)
ν(**C-O**)	1110 (s)
ν(**C-O**)	1047 (m)
δ$_{out\ of\ plane}$ (C=O)**O-H**	965 (s)

Table S1. FTIR assignment of pure NDPM ligand.

**Band**	**Wavenumber (cm^{-1})**
ν_{as} (N–H)$_{secondary\ amine}$	3300 (m)
ν_{s} (**C=O**)$_{amide}$	1640 (s)
δ (N–H)$_{amide}$	1590 (s)
ν_{as} (N–O)$_{nitro}$	1525 (s)
ν_{s} (N–O)$_{nitro}$	1395 (s)
ν_{s} (C–N)	1288 (s)
Aromatic C–O	1233 (s)
ν(**C–OH**)$_{aromatic}$	1195 (s)
ν(**C–O**)$_{aromatic}$	1105 (s)

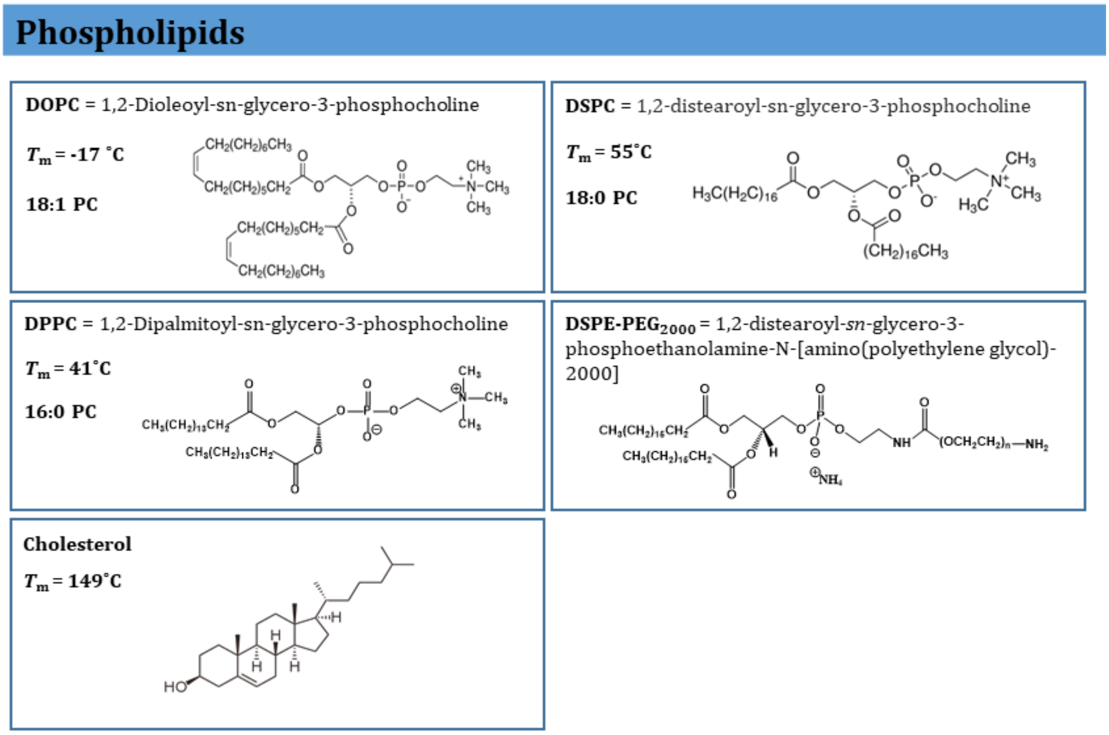

Figure S2. Structure, chemical name and phase transition temperature (T_m) of used lipids in this study.

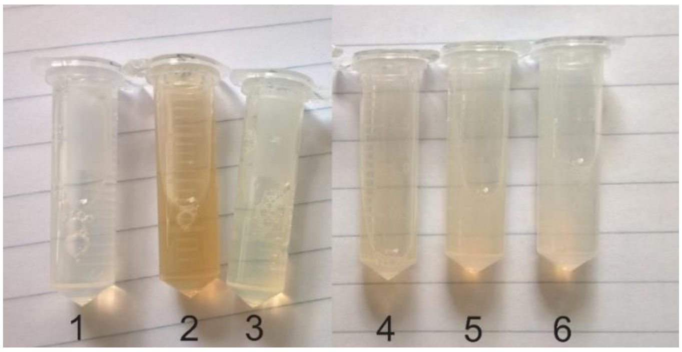

Figure S3. MLs prepared using lipid film hydration and extrusion, using with different lipids composition. 1=DOPC/DSPE-PEG$_{2000}$, 2=DSPC/DSPE-PEG$_{2000}$, 3=DPPC/DSPE-PEG$_{2000}$, 4=DOPC/Chol/DSPE-PEG$_{2000}$, 5=DSPC/Chol/DSPE-PEG$_{2000}$, 6=DPPC/Chol/DSPE-PEG$_{2000}$. The same concentration of IO NPs (0.3 mL of 1mg/mL) was used for the sample preparation.

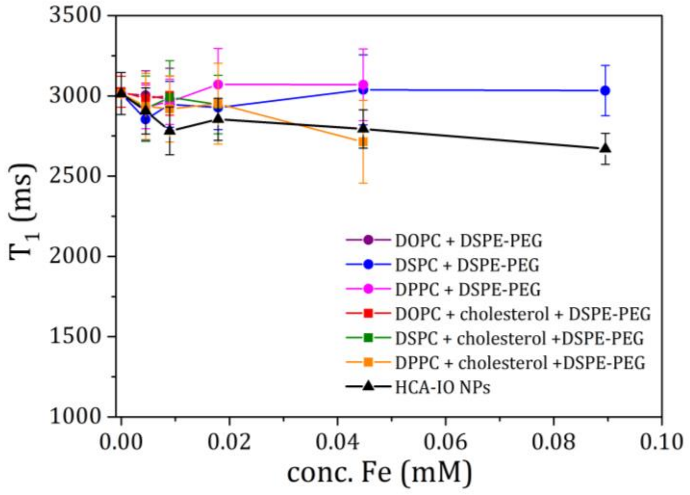

Figure S4. T_1 relaxation times of MLs and HCA-coated IO NPs.

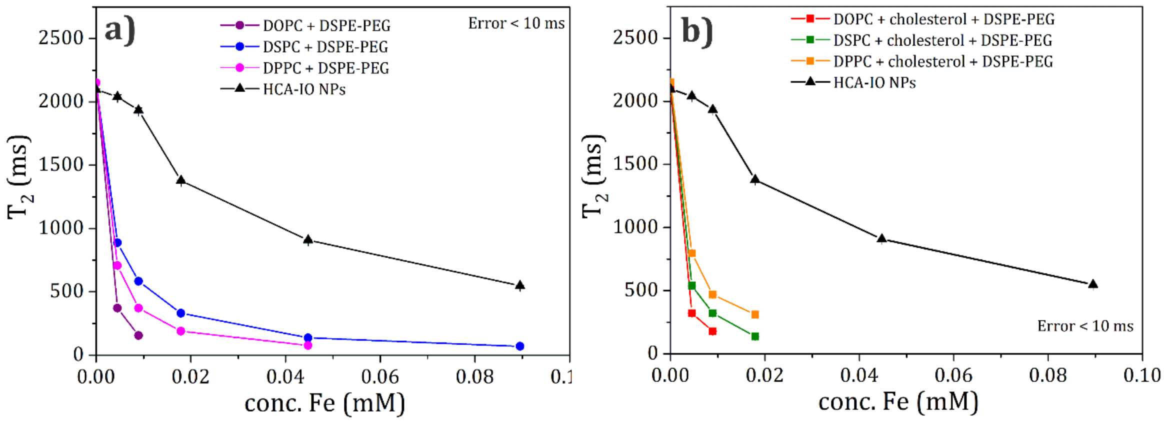

Figure S5. T_2 relaxation times of MLs and HCA-coated IO NPs. T_2 relaxation times of a) non-cholesterol and b) cholesterol-containing MLs. The range of concentration measured was limited by the initial Fe content in the samples.

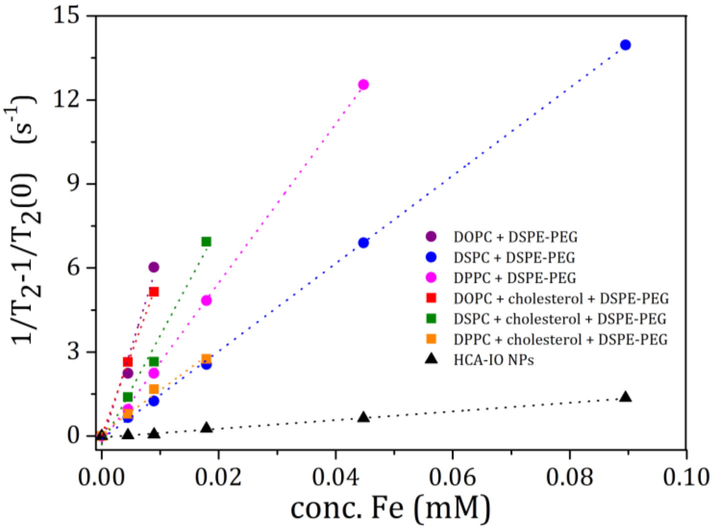

Figure S6. Graph showing inverse transverse $1/T_2$ - $1/T_2(0)$ relaxation rate increase for MLs and HCA-coated IO NPs.

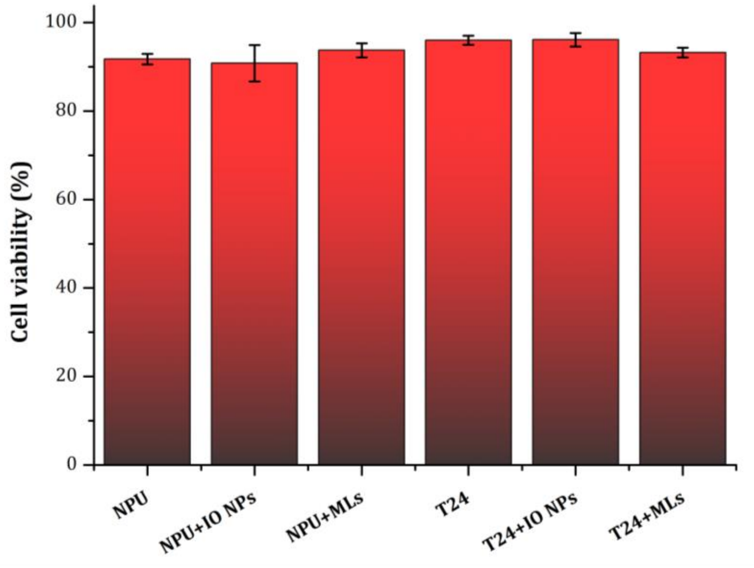

Figure S7. Normal NPU, and cancerous T24 cells were incubated with HCA-IO NPs or MLs for 24 h and cell viabilities (n=4) were determined using trypan-blue staining.

Literature overview for magneto-liposomes as MRI contrast agents

Interestingly, from all the reports investigating of MLs for MRI applications either with IO NPs in the liposomal core[3–10] or in the bilayer[11,12], no *in vitro* MRI studies can be found. They were testing either MLs suspensions only (*ex vitro*)[4–6,8,12] or performing directly *in vivo* imaging[3,7,9–11] without prior *in vitro* investigation. Detailed list of above-mentioned references with the summary of their experimental conditions (size of IO NPs and their location in the liposomes, liposomal formulation, concentration range used for the determination of r_2, maximum r_2 and *in vivo* conditions) can be found in the **Table S3**.

Table S3. List of all studies using MLs for MRI applications with the summary of their experimental data (size of IO NPs and their location in the liposomes, liposomal formulation, concentration range used for the determination of r_2, max r_2 and in vivo conditions).

Ref. (NPs location)	*Ex vitro* i.e. suspension only (formulation, Fe conc. range and max r_2)	*In vivo* (conc. used and administration route)
[3] **Shen et al.**<br>14 nm IO NPs in the core	DPPC/Chol (80/20 mol%)<br>0 - 0.8 mM<br>r_2 = 20.49	Intravenously 200 µL of MLs with $Fe3O_4$ concentration of 2 mg/mL + magnet next to the tumor. Tumor appeared 59% darker.
[4] **Carvalho et al.**<br>6 nm IO NPs core	Soybean PC + Cholesterol<br>Fe 0 – 2.5 mM<br>max r_2 = 143.69 without Chol	/
[5] **Skouras et al.**<br>size not specified, core	Conc. range not specified, only relaxivities shown	/
[6] **Garnier et al.**<br>7 IO NPs in the core	DOPC/Chol (75/25 mol%)<br>0 - 0.8 mM<br>max r_2 = 323	/
[7] **Marie et al.**<br>13 nm IO NPs in the core	EPC/DSPE-PEG2000/Rho-PE = 94/5/1<br>0 – 0.2mM<br>r_2 = 259	Intravenous injection 200µL MLs (122.5 µmoles lipids and 533 µmoles iron oxide per kg) + magnet next to the tumor<br>Tumor appears darker
[8] **Faria et al.**<br>11 nm IO NPs in the core	SPC/Chol = 1/0.5<br>no conc. ranges	/

	Agar phantoms – T2 images slightly darker	
[9]**Martina et al.**<br>17 nm IO NPs in the core	EPC/DSPE-PEG2000 (95/5)<br>0.02 -10mM<br>Max r_2 = 130	Intravenous of 200 μL of MLs (20 mM total lipid and 25 mM Fe). Tumor 22% brighter on T_1 image-
[10] **Béalle et al.**<br>7 nm IO NPs in the core	DPPC/DSPC (90/10)<br>0 - 1 mM<br>max r_2 = 267.9	DPPC/DSPC/Rhod-PE/DSPE-PEG (94/10/1/5)<br>100 μL of MLs with 0.1 mM Fe retro-orbital venous sinus injection + magnet next to the tumor. Black dots in the tumor
[11] **Guo et al.**<br>4 nm IO NPs in the bilayer	DPPC/Chol/SA/DSPE-MPEG2000-MTX<br>0-1 mM T2-weighted images<br>r_2 = 60.06	Intravenously 0.2 mL of 2 mg/kg (DOX eq. dose) + tumor next to the tumor.<br>Colored T2 images
[12] **Martínez-González et al.**<br>Hydrophobic 5 nm IO NPs in the bilayer<br>Hydrophilic NPS not in the liposomes, but forming branched-linear clusters	DMPC, DMPC/Chol, DMPC-PS, DOPC, DOPC/Chol, DOPC-PS<br>0 – 0.12 mM<br>Max r_2=995 for DOPC-PS	/

References

1. Niebel, T.P.; Heiligtag, F.J.; Kind, J.; Zanini, M.; Lauria, A.; Niederberger, M.; Studart, A.R. Multifunctional microparticles with uniform magnetic coatings and tunable surface chemistry. *RSC Adv.* **2014**, *4*, 62483–62491.

2. Bixner, O.; Lassenberger, A.; Baurecht, D.; Reimhult, E. Complete Exchange of the Hydrophobic Dispersant Shell on Monodisperse Superparamagnetic Iron Oxide Nanoparticles. *Langmuir* **2015**, *31*, 9198–9204.

3. Shen, S.; Huang, D.; Cao, J.; Chen, Y.; Zhang, X.; Guo, S.; Ma, W.; Qi, X.; Ge, Y.; Wu, L. Magnetic liposomes for light-sensitive drug delivery and combined photothermal–chemotherapy of tumors. *J. Mater. Chem. B* **2019**, *7*, 1096–1106.

4. Carvalho, A.; Goncalves, M.C.; Martins, M.B.F.; Meixedo, D.; Feio, G. Relaxivities of magnetoliposomes: The effect of cholesterol. *Magn. Reson. Imaging* **2013**, *31*, 610–612.

5. Skouras, A.; Mourtas, S.; Markoutsa, E.; De Goltstein, M.-C.; Wallon, C.; Catoen, S.; Antimisiaris, S.G. Magnetoliposomes with high USPIO entrapping efficiency, stability and magnetic properties. *Nanomedicine Nanotechnology, Biol. Med.* **2011**, *7*, 572–579.

6. Garnier, B.; Tan, S.; Miraux, S.; Bled, E.; Brisson, A.R. Optimized synthesis of 100 nm diameter magnetoliposomes with high content of maghemite particles and high MRI effect. *Contrast Media Mol. Imaging* **2012**, *7*, 231–239.

7. Marie, H.; Lemaire, L.; Franconi, F.; Lajnef, S.; Frapart, Y.-M.; Nicolas, V.; Frebourg, G.; Trichet, M.; Menager, C.; Lesieur, S. Superparamagnetic Liposomes for MRI Monitoring and External Magnetic Field-Induced Selective Targeting of Malignant Brain Tumors. *Adv. Funct. Mater.* **2015**, *25*, 1258–1269.

8. Faria, M.R.; Cruz, M.M.; Gonçalves, M.C.; Carvalho, A.; Feio, G.; Martins, M.B.F. Synthesis and characterization of magnetoliposomes for MRI contrast enhancement. *Int. J. Pharm.* **2013**, *446*, 183–190.

9. Martina, M.S.; Fortin, J.P.; Ménager, C.; Clément, O.; Barratt, G.; Grabielle-Madelmont, C.; Gazeau, F.; Cabuil, V.; Lesieur, S. Generation of superparamagnetic liposomes revealed as highly efficient MRI contrast agents for in vivo imaging. *J. Am. Chem. Soc.* **2005**, *127*, 10676–10685.

10. Béalle, G.; Di Corato, R.; Kolosnjaj-Tabi, J.; Dupuis, V.; Clément, O.; Gazeau, F.; Wilhelm, C.; Ménager, C. Ultra magnetic liposomes for MR imaging, targeting, and hyperthermia. *Langmuir* **2012**, *28*, 11834–11842.

11. Guo, Y.; Zhang, Y.; Ma, J.; Li, Q.; Li, Y.; Zhou, X.; Zhao, D.; Song, H.; Chen, Q.; Zhu, X. Light/magnetic hyperthermia triggered drug released from multi-functional thermo-sensitive magnetoliposomes for precise cancer synergetic theranostics. *J. Control. Release* **2018**, *272*, 145–158.

12. Martínez-González, R.; Estelrich, J.; Busquets, M.A. Liposomes Loaded with Hydrophobic Iron Oxide

Nanoparticles: Suitable T_2 Contrast Agents for MRI. *Int. J. Mol. Sci.* **2016**, *17*, 1209.
